# Higher dietary acid load is associated with an increased risk of metabolic syndrome

**DOI:** 10.1038/s41598-023-48429-2

**Published:** 2023-12-13

**Authors:** Najmeh Seifi, Hamidreza Rahimi, Glareh Koochakpoor, Amin Zarei, Reza Assaran Darban, Gordon A. Ferns, Majid Ghayour-Mobarhan

**Affiliations:** 1 International UNESCO Center for Health-Related Basic Sciences and Human Nutrition, Mashhad University of Medical Sciences, Mashhad, Iran; 2 School of Nursing and Allied Medical Sciences, Maragheh University of Medical Sciences, Maragheg, Iran; 3 Department of Biology, Mashhad Branch, Islamic Azad University, Mashhad, Iran; 4 Department of Biology, Mashhad Branch, Islamic Azad University, Mashhad, Iran; 5 Division of Medical Education, Brighton &amp; Sussex Medical School, Falmer, Brighton, Sussex UK; 6 Metabolic Syndrome Research Center, Mashhad University of Medical Sciences, Mashhad, 99199-91766 Iran

**Keywords:** Diseases, Endocrinology, Medical research, Risk factors

## Abstract

There have been inconsistent reports regarding the association between dietary acid load and Metabolic Syndrome (MetS). We aimed to investigate the association between dietary acid load and MetS in an Iranian adult population. In this cross-sectional study, 1945 participants aged 35–65 years were recruited from MASHAD cohort study. Dietary intakes were assessed using a 24-h dietary recall. Diet-based acidity was assessed as the net endogenous acid production (NEAP), potential renal acid load (PRAL), and dietary acid load (DAL). To define MetS, the International Diabetes Federation (IDF) criteria were used. Multivariable logistic regression models were applied to determine the association between diet-based acid load scores and MetS. Participants' mean age and BMI were 47.13 ± 7.78 years and 27.57 ± 4.48 kg/m^2^, respectively. Around 57% of the population was female. Overall, 31.9% had MetS. According to the full-adjusted model, there was a significant association between higher quartiles of PRAL, NEAP, and DAL and MetS (Q4 PRAL; OR (95%CI) 1.42(1.05–1.91), Q4 NEAP; OR (95%CI) 1.48(1.11–1.98), Q4 DAL; OR (95%CI) 1.44(1.05–1.91)). This study showed a significant positive association between different dietary acid load indicators (PRAL, NEAP, and DAL) and odds of MetS among Iranian adults.

## Introduction

Metabolic syndrome (MetS) is a clustering of cardiovascular risk factors, including hyperinsulinemia, impaired fasting glucose, type 2 diabetes mellitus, abdominal obesity, dyslipidemia, hypertension, and low levels of high-density lipoprotein cholesterol (HDL-c)^1^. The diagnosis and treatment of MetS are clinically important because of its potentially harmful effects on the risk of developing diabetes mellitus, cardiovascular disease, and mortality^2,3^ and its increasing prevalence over the last three decades^4^. According to different diagnostic criteria, the prevalence of metabolic syndrome in Iran was reported to be 30.8–44.6%^5^. While lifestyle modification, such as increased physical activity and a healthy diet, has been proposed as the most effective strategy to manage MetS^6^, there is no consensus on the most effective diet on all its clinical indicators. Therefore, finding the best dietary strategies to combat this heterogeneous disease has been a concern for many researchers.

In recent years, increased hydrogen ion body burden has attracted attention due to more adherence to the Western dietary pattern characterized by high-protein foods and insufficient fruit and vegetable intake has attracted attention^7^. Metabolic acidosis is associated with an elevated risk of morbidity and mortality because of its unfavorable health effects, such as abdominal obesity, insulin resistance, high cortisol levels, high triglyceride (TG) levels, and increased BP^8^. Net endogenous acid production (NEAP), potential renal acid load (PRAL), and dietary acid load (DAL) are commonly used indicators to measure dietary acid load^9,10^.

Several studies have been conducted to determine the association between dietary acid load scores and the risk of metabolic syndrome and its components. Nevertheless, their results have been contradictory. Two cross-sectional studies indicated no significant association between DAL and odds of MetS in Iranian women^11,12^. Whereas, in another study performed on 14,105 women and 14,042 men (aged 35–69 years) in Japan, higher scores of NEAP were associated with significantly increased odds of MetS, independent of nutrient pattern or carbohydrate intake^13^. Two recent studies on older adults also showed a significant positive association between dietary acid load and MetS^14,15^. A meta-analysis in 2023 also revealed that higher DAL scores were associated with elevated risk of MetS. However, there was significant heterogeneity across the included studies^16^. Therefore, the following study was administered to investigate the association of three dietary acidity indicators (PRAL, NEAP, and DAL) and the odds of MetS among the Iranian population, considering gender differences.

## Methods

### Study design and population

This cross-sectional study was conducted on adult participants aged 35–65 recruited for the Mashhad Stroke and Heart Sclerotic Disorders (MASHAD) study. Demographic, anthropometric, biochemical, and blood pressure parameters were determined at baseline. Pregnant or lactating women and participants with incomplete dietary intake data or over-reporting (> 4200 kcal) and under-reporting of energy intake (< 800 kcal) were excluded; the final analysis was done on 1945 subjects. The Human Research Ethics Committee of Medical School, Mashhad University of Medical Sciences (MUMS) approved the study protocol. All of our participants provided their consent, which was informed and in written form.

### Anthropometric measurements

As previously described, standard protocols were applied to measure height, weight, and waist circumference (WC)^17^. Body mass index (BMI) was quantified as weight in kilograms divided by the square of height in meters.

### Blood pressure measurement

After a 15-min rest, the blood pressure was evaluated in the seated position, with the participant’s arm at the heart level, applying a standard mercury manometer. The evaluation was repeated after 15 min. The mean value of the two measurements was regarded as the individual’s blood pressure.

### Physical activity level (PAL) measurement

Physical activity was measured using a validated questionnaire as described before^18^. The physical activity questions were categorized into time spent on work activities, non-work activities, and in bed. The integrated energy index (IEI) ranged from 1.61 for inactive to 4.39 for active times. PAL < 1.5, 1.5–2, and > 2 were considered inactive, moderately active, and active, respectively.

### Biochemical measurements

After a 12-h fasting, 10 ml of peripheral vein blood was taken and centrifuged for 30–45 min at room temperature. Then, serum aliquots were kept at − 80 °C. Fasting plasma glucose (FPG), serum triglyceride (TG), high-density lipoprotein-cholesterol (HDL-C), low-density lipoprotein-cholesterol (LDL-C), and total cholesterol were measured with the application of commercial kits supplied by Pars Azmoon (Tehran, Iran), and a BT-3000 autoanalyzer (Biothecnica, Italy). Further details are presented elsewhere^17^.

### Dietary intakes

Dietary intakes were assessed using a 24- hour dietary recall in a face-to-face interview and then statistical modeling to estimate distributions of usual intake^19^. To analyze the energy and nutrient intakes Diet plan 6 software (Forestfield Software Ltd., Horsham, West Sussex, UK) was used. Dietary intakes were energy-adjusted using the method of residuals^20^. We constructed the food groups as follows: Fruits and vegetables: fresh fruits, dried fruits, green leafy vegetables, starch vegetables, and other vegetables; dairy: yogurt, milk, yogurt drink, cheese, ice-cream, and cream; white meats and egg: fishes, poultry meat, eggs; red meats: meats: processed meat and red meat; whole grain: whole-grain bread; refined grain: white bread, pasta, biscuits, rice; sugar-sweeten beverages (SSBs) and sweets: honey, cakes and cookies, sugar, sugar loaf, industrial fruit juices, soft drinks, malt beverages, and chocolate.

### Dietary acid load

Dietary acid load was estimated using three indicators. The net endogenous acid production (NEAP), the potential renal acid load (PRAL), and dietary acid load (DAL). The following formulas were used^21–24^:

- NEAP (mEq/day) = 54.5 × [protein intake (g/d) /K intake (*mEq*/d)] − 10.2,
- PRAL (mEq/day) = 0.49 × protein (g day) + 0.037 × phosphorus (mg day) − 0.021 × potassium ( mg day) − 0.026 × magnesium (mg day) − 0.013 × calcium (mg day),
- DAL (mEq/day) = PRAL + (body surface area [m2] × 41[mEq/day]/1.73 m^2^),
- Body surface area was calculated as: 0.024265 × Height (cm)^0.3964^ × Weight (kg).

### Metabolic syndrome (MetS)

To define MetS, the international diabetes federation (IDF) criteria were used. It includes a waist circumference > 94 cm in men and > 80 cm in women, plus any two of the followings: (1) serum TG ≥ 150 mg/dl or taking medications for this type of dyslipidemia, (2) HDL < 40 mg/dl in men, and < 50 mg/dl in women, (3) FPG ≥ 100 mg/dl or having type 2 diabetes (4) systolic blood pressure (SBP) ≥ 130 mmHg or diastolic blood pressure (DBP) ≥ 85 or being on treatment for hypertension (HTN).

### Statistical analysis

Statistical analysis was carried out by the Statistical Package for the Social Science version 25.0 (SPSS, Chicago, IL). The Kolmogorov–Smirnov test checked the normal distribution of variables; one-way ANOVA and Chi-square were used to compare variables between different quartiles of DAL. Logistic regression analysis was applied to investigate the association between DAL and metabolic syndrome while controlling the effect of age, BMI, sex, energy intake, food groups (fruit and vegetables, Dairy, SSBs and sweets), and physical activity level.

### Ethics approval and consent to participate

All experiments were performed in accordance with the declaration of Helsinki and Mashhad University of Medical Sciences ethical guidelines and regulations. The research protocol was approved by the School of Medicine, Mashhad University of Medical Sciences, Biomedical Research Ethics Committee (IR.MUMS.MEDICAL.REC.1398.228). All participants signed a written inform consent before participating in the study.

## Results

One thousand nine hundred and forty-five individuals were included in the final analysis. The mean age of participants was 47.13 ± 7.78 years. Around 57% of the population were female. Baseline characteristics of study participants is presented by categories of DAL in Table 1.

**Table 1:** Baseline characteristics of study participants by categories of DAL.

	N = 1945	Q1 (n = 487)	Q2 (n = 486)	Q3 (n = 486)	Q4 (n = 486)	p-value	P-trend
PRAL (mEq/day)	5.61 ± 20.50	− 18.9 ± 15.64	0.69 ± 3.36	11.21 ± 3.00	29.49 ± 13.73	< 0.001	< 0.001
NEAP	44.63 ± 17.72	26.42 ± 6.65	38.68 ± 6.37	49.41 ± 10.27	64.06 ± 17.30	< 0.001	< 0.001
DAL (mEq/day)	7.08 ± 20.51	17.44 ± 15.62	2.15 ± 3.36	12.67 ± 3.00	31.00 ± 13.74	< 0.001	< 0.001
Age (year)	47.13 ± 7.78	47.32 ± 7.58	47.31 ± 7.67	47.52 ± 8.03	46.37 ± 7.83	0.093	0.094
Sex							
Male, n (%)	823 (42.3)	(156)	(170)	(207)	(281)	< 0.001	–
Female, n (%)	1122 (57.5)	(322)	(316)	(279)	(205)		
BMI	27.57 ± 4.48	27.56 ± 4.27	27.89 ± 4.68	27.46 ± 4.56	27.38 ± 4.39	0.307	0.283
Waist Circumference (cm)	94.04 ± 11.98	93.46 ± 11.75	94.31 ± 12.04	93.95 ± 12.05	94.40 ± 12.09	0.598	0.308
LDL-C	116.94 ± 31.89	115.97 ± 31.66	117.61 ± 32.82	116.66 ± 31.29	117.51 ± 31.87	0.836	0.572
HDL-C	42.20 ± 9.53	42.14 ± 9.46	43.21 ± 9.79	42.31 ± 9.33	41.12 ± 9.43	0.008	0.041
Triglyceride	127.39 ± 77.27	126.85 ± 68.72	130.01 ± 82.75	125.21 ± 80.49	127.53 ± 76.59	0.810	0.861
Total Cholesterol	187 ± 34.69	186.69 ± 33.99	189.56 ± 35.80	185.32 ± 35.08	186.45 ± 33.83	0.269	0.482
FPG (mg/dl)	82.73 ± 21.04	82.02 ± 23.81	82.23 ± 21.37	83.27 ± 17.96	83.41 ± 20.65	0.649	0.224
SBP (mmHg)	118.19 ± 15.34	118 ± 15.12	117.85 ± 15.97	119.29 ± 15.85	117.64 ± 14.45	0.334	0.905
DBP	77.77 ± 10.40	77.51 ± 10.27	77.68 ± 10.31	78.76 ± 10.65	77.11 ± 10.32	0.084	0.956
Smoking status (%)							
Non-smoker	71.2	73.5	71.4	71	68.9	0.661	–
Current-smoker	20.4	17.9	20.8	21.4	21.6		
Ex-smoker	8.4	8.6	7.8	7.6	9.5		
Job (%)							
Student	0.1	0	0.2	0.2	0	< 0.001	–
Employed	38.5	35.1	32.3	36.4	50.2		
Unemployed	51.8	54.6	58	53.7	40.9		
Retired	9.5	10.3	9.3	9.7	8.8		
Physical activity	1.57 ± 0.27	1.59 ± 0.27	1.59 ± 0.25	1.59 ± 0.28	1.53 ± 0.29	< 0.001	0.001
Metabolic syndrome (%)	31.9	35.1	31.1	33.1	28.4	0.132	–

*BMI* body mass index, *DAL* dietary acid load, *DBP* diastolic blood pressure, *FPG* fasting plasma glucose, *HDL* high-density lipoprotein cholesterol, *LDL* low-density lipoprotein cholesterol, *NEAP* net endogenous acid production, *PRAL* potential renal acid load, *SBP* systolic blood pressure.

PRAL, NEAP and DAL score were significantly different in DAL quartiles, with an increasing trend (p < 0.001). There was no significant difference in age, BMI, and WC among different categories of DAL. Participants assigned to the highest category of DAL had significantly lower levels of HDL cholesterol. There was also a significantly decreasing trend in HDL cholesterol levels (p = 0.04). Patients in the higher quartiles of DAL were more prone to have lower physical activity levels (p = 0.001). For other characteristics, except job status, there was no difference in DAL categories.

The dietary intake of participants for quartiles of DAL is presented in Table 2. Among food groups, DAL was negatively associated with fruits and vegetables (p < 0.001) and dairy (p = 0.003) but positively associated with SSBs and sweets (p = 0.002). Regarding energy and macronutrients, DAL was positively associated with energy, protein, total fat, MUFA and saturated fatty acids intakes (p < 0.001), while it was negatively associated with carbohydrates and fiber intake (p < 0.001). There was no significant association in PUFA intakes in the DAL quartiles. Considering dietary intakes of micronutrients, DAL was negatively associated with sodium, potassium, calcium, magnesium, and iron; however, it was positively associated with phosphorus and zinc intake.

**Table 2:** Food groups, macronutrient, and micronutrient intake of study participants by categories of DAL.

	Q1 (n = 487)	Q2 (n = 486)	Q3 (n = 486)	Q4 (n = 486)	p-value	P trend
Food groups						
Whole grain (g/day)	149.04 ± 116.23	136.57 ± 116.75	150.69 ± 126.62	145.09 ± 140.05	0.260	0.954
Refined grain(g/day)	180.43 ± 105.75	187.59 ± 128.09	184.66 ± 96.29	179.65 ± 93.66	0.614	0.807
Fruits and vegetables(g/day)	499.79 ± 263.20	465.09 ± 267.22	426.65 ± 279.38	417.70 ± 279.37	< 0.001	< 0.001
Dairy(g/day)	364.62 ± 198.67	348.08 ± 208.34	315.70 ± 185.56	333.392 ± 217.87	0.001	0.003
Red meat(g/day)	44.43 ± 37.08	45.21 ± 45.31	40.26 ± 29.19	43.88 ± 36.94	0.121	0.385
White meat and egg(g/day)	62.99 ± 35.46	65.74 ± 48.09	64.14 ± 35.08	65.97 ± 33.09	0.465	0.307
SSBs and sweets(g/day)	139.31 ± 113.47	153.00 ± 133.03	152.30 ± 145.45	168.85 ± 164.10	0.012	0.002
Macronutrients and energy						
Energy (Kcal/day)	1879.09 ± 685.55	1681.79 ± 585.45	1763.18 ± 616.35	2165.51 ± 663.71	< 0.001	< 0.001
Carbohydrate (g/day)	257.77 ± 58.15	248.97 ± 44.48	240.45 ± 47.14	222.51 ± 55.49	< 0.001	< 0.001
Protein(g/day)	61.72 ± 16.86	64.94 ± 15.35	65.75 ± 14.75	75.28 ± 25.02	< 0.001	< 0.001
Fiber (g/day)	23.08 ± 9.27	17.99 ± 7.19	14.48 ± 6.89	12.49 ± 8.13	< 0.001	< 0.001
MUFA (mg/day)	17.24 ± 7.69	18.74 ± 5.44	20.09 ± 5.56	22.04 ± 7.58	< 0.001	< 0.001
PUFA (mg/day)	23.54 ± 13.56	23.13 ± 10.60	24.29 ± 12.10	23.91 ± 14.19	0.525	0.373
Saturated fat (mg/day)	16.85 ± 7.36	18.17 ± 6.20	19.03 ± 6.98	19.73 ± 8.43	< 0.001	< 0.001
Fat (g/day)	66.59 ± 23.46	68.86 ± 17.94	72.02 ± 19.94	75.19 ± 23.60	< 0.001	< 0.001
Micronutrients						
Sodium (mg/day)	3999.55 ±	2742.65 ± 5684.08	2307.60 ± 3000.26	3027.63 ± 7673.23	0.004	0.02
Potassium (mg/day)	3623.958.93	2776.13 ± 625.84	2418.90 ± 595.20	2157.42 ± 713.74	< 0.001	< 0.001
Calcium (mg/day)	922.66 ± 350.25	817.58 ± 278.47	801.01 ± 305.15	774.15 ± 334.87	< 0.001	< 0.001
Magnesium (mg/day)	291.63 ± 98.33	248.35 ± 78.72	221.15 ± 70.98	216.93 ± 95.37	< 0.001	< 0.001
Phosphorus (mg/day)	1251.16 ± 334.51	1219.78 ± 279.07	1253.40 ± 305.12	1398.54 ± 363.89	< 0.001	< 0.001
Iron (mg/day)	12.39 ± 5.37	11.22 ± 4.55	9.97 ± 4.40	9.72 ± 5.18	< 0.001	< 0.001
Copper (mg/day)	2.01 ± 0.77	1.96 ± 0.61	1.88 ± 0.95	2.15 ± 2.48	0.02	0.19
Zinc (mg/day)	8.71 ± 2.64	9.09 ± 2.59	9.04 ± 4.40	9.54 ± 3.42	< 0.001	< 0.001

*MUFA* mono-unsaturated fatty acids, *PUFA* polyunsaturated fatty acids.

Regarding the association between MetS and DAL scores, odds ratios (ORs) and 95% confidence intervals (CI) are shown in Table 3. In the crude model, higher PRAL, NEAP, and DAL scores were significantly associated with higher odds of metabolic syndrome. In model 1, adjusted for sex, age, BMI, energy intakes, and physical activity, NEAP score was significantly associated with higher odds of metabolic syndrome, as the risk of MetS in the highest quartile of NEAP was 42% greater than the lowest quartile (OR = 1.42, p = 0.014). In model 1, the odds of MetS in the highest quartile of DAL were 1.36 times greater than the lowest quartile (p = 0.039). When further adjusted for dietary intakes of dairy, vegetables and fruits, SSBs and sweets, the highest PRAL, NEAP, and DAL quartiles were significantly associated with increased odds of metabolic syndrome compared to the lowest quartile.

**Table 3:** Logistic regression analysis models for the association between metabolic syndrome and quartiles of PRAL, NEAP, and DAL.

Variable			PRAL				NEAP				DAL			
			Q1	Q2	Q3	Q4	Q1	Q2	Q3	Q4	Q1	Q2	Q3	Q4
Metabolic syndrome	Crude	OR	1	1.18	1.13	1.35	1	1.16	1.14	1.48	1	1.20	1.09	1.37
		95% CI		0.90–1.54	0.86–1.47	1.03–1.77		0.91–1.55	0.94–1.61	1.08–1.86		0.91–1.56	0.84–1.42	1.04–1.79
		p-value	-	0.221	0.366	0.028	-	0.232	0.286	0.003	-	0.180	0.499	0.023
	Model 1	OR	1	1.32	1.14	1.34	1	1.24	1.04	1.42	1	1.34	1.11	1.36
		95% CI		0.99–1.75	0.85–1.51	1.00–1.80		0.97–1.72	0.85–1.51	1.06–1.90	–	1.00–1.78	0.83–1.47	1.01–1.83
		p-value	–	0.058	0.366	0.050	–	0.116	0.783	0.014	–	0.047	0.479	0.039
	Model 2	OR	1	1.36	1.19	1.42	1	1.27	1.06	1.48	1	1.379	1.166	1.44
		95% CI	–	1.02–1.81	0.90–1.59	1.05–1.91		0.96–1.67	0.80–1.40	1.11–1.98		1.02–1.82	0.86–1.52	1.05–1.91
		p-value	–	0.036	0.214	0.021		0.089	0.646	0.006	–	0.029	0.292	0.016

Crude: unadjusted, Model1: adjusted for age, BMI, Model2: Model1 + Physical activity level, education, smoking status, energy intake.

*DAL* dietary acid load, *NEAP* net endogenous acid production, *PRAL* potential renal acid load.

In Table 4, the results of crude and multi-variable adjusted ORs and 95% CI for the association between MetS and DAL scores, stratified by sex, are presented. In the male population, higher PRAL, NEAP, and DAL scores were significantly associated with higher odds of metabolic syndrome. In the full-adjusted model, the odds of MetS in the PRAL, NEAP, and DAL highest quartiless were 1.84, 1.84, and 1.86 times greater than the lowest quartiles, respectively. In females, no significant association was observed between any indices of dietary acid load and MetS.

**Table 4:** Logistic regression analysis models for the association between metabolic syndrome and quartiles of PRAL, NEAP, and DAL categorized by sex.

Variable		PRAL				NEAP				DAL			
		Q1	Q2	Q3	Q4	Q1	Q2	Q3	Q4	Q1	Q2	Q3	Q4
Male	Crude	1	1.03 (0.65–1.62)	1.19 (0.77–1.85)	1.52(0.99–2.32)	1	1.03 (0.65–1.61)	1.29 (0.85–1.98)	1.54 (0.99–2.40)	1	1.04 (0.66–1.65)	1.16 (0.75–1.81)	1.52 (1.00–2.33)
	Model 1	1	0.97 (0.58–1.62)	1.26 (0.76–2.09)	1.74 (1.08–2.83)	1	0.98 (0.60–1.62)	1.26 (0.78–2.04)	1.72 (1.05–2.83)	1	0.88 (0.59–1.66)	1.22 (0.74–2.01)	1.76 (1.09–2.85)
	Model 2	1	0.98 (0.59–1.65)	1.29 (0.77–2.15)	1.84 (1.14–2.99)	1	1.02 (0.62–1.70)	1.30 (0.80–2.09)	1.84 (1.11–3.06)	1	1.01 (0.60–1.69)	1.24 (0.75–2.06)	1.86 (1.15–3.02)
Female	Crude	1	1.27 (0.91–1.77)	1.06 (0.76–1.48)	1.08 (0.75–1.56)	1	1.29 (0.93–1.80)	1.13 (0.80–1.59)	1.25 (0.88–1.79)	1	1.29 (0.93–1.79)	1.03 (0.73–1.43)	1.11 (0.77–1.60)
	Model 1	1	1.46 (1.03–2.07)	1.07 (0.75–1.51)	1.06 (0.72–1.57)	1	1.42 (1.00–2.01)	1.03 (0.71–1.47)	1.28 (0.88–1.84)	1	1.48 (1.04–2.09)	1.03 (0.73–1.47)	1.09 (0.74–1.60)
	Model 2	1	1.52 (0.11–2.16)	1.14 (0.80–1.62)	1.13 (0.76–1.67)	1	1.47 (1.03–2.08)	1.09 (0.76–1.58)	1.38 (0.95–2.01)	1	1.53 (1.08–2.18)	1.11 (0.78–1.57)	1.15 (0.78–1.71)

Crude: unadjusted, Model1: adjusted for age, BMI, Model2: Model1 + Physical activity level, education, smoking status, energy intake.

*DAL* dietary acid load, *NEAP* net endogenous acid production, *PRAL* potential renal acid load.

## Discussion

In the present cross-sectional study that included 1945 Iranian adults, the odds ratio (OR) of MetS in subjects in the highest quartiles of PRAL, NEAP and DAL compared to the lowest were 42%, 48%, and 44% higher, respectively. Further analysis, splitting the population by sex, showed that in the male population, higher PRAL, NEAP, and DAL scores were significantly associated with increased odds of metabolic syndrome, while in females, there was no significant association. To the best of our knowledge, no previous study has investigated the relationship between three different indicators of dietary acid–base load (DAL, NEAP, and PRAL) and the risk of metabolic syndrome in Iranian adults considering sex differences. Previous studies have been limited to women^11,25^, adolescents^15^, and elderly^26^, or they have only considered the association of two indices with MetS in the general population^12^. Each indicator has a different formula, limitations and advantages^27^. Therefore, using three indicators provides more reliable results.

The literature review conducted in this field showed that the results of the existing studies are very conflicting, and the gender and age differences in the study populations may justify these differences. Contrary to the results of our study, in Mohammadi Fard et al.'s study, carried out on 1430 Iranian adults (both men and women), there was no significant relationship between the acid load of the diet and metabolic syndrome. However, higher NEAP was associated with higher fasting blood glucose^12^. Two other studies on Iranian women also showed no significant association between dietary acid load and MetS^11,28^, which is in accordance with our results. On the other hand, two other studies on Iranian elders and adolescents showed that higher NEAP was significantly associated with higher odds of MetS. The Japan Multi-Institutional Collaborative Cohort Study results also showed that NEAP was positively associated with MetS^13^. Although there was significant evidence of heterogeneity across studies, a recent meta-analysis revealed that there was a significant linear association between NAEP and PRAL with MetS. In subgroup analysis, the association of NEAP and MetS was supported in the studies of both genders but not in women or men subgroups^16^.

As we previously described, differences in the age, gender, and BMI of the study population may justify the differences in the results. Besides, the differences may also be attributed to the diet’s acidity level. The mean PRAL and NEAP levels in our study (5.61 and 44.63 mEq/d, respectively) were lower than the mean PRAL and NEAP levels in the Mozaffari et al. study (9.61 and 47.46 mEq/d, respectively)^11^. Nevertheless, regarding NEAP level, we are similar to other studies that declared significant associations with MetS^13,14^. These differences in the participants' exposure to dietary acid–base can make it difficult to compare the results. On the other hand, there are food items that, although generally considered acidogenic, their consumption can have a protective effect on the occurrence of MetS by several other mechanisms. Among these food items, we can mention fish, dairy products, and cereal^29,30^. Therefore, consuming these foods can be confounding and change the relationship between DAL and metabolic syndrome. The variation in MetS definition may also influence the results. The subgroup analysis in the meta-analysis by Al-hawary et al. showed that the association of NEAP with MetS was declared in studies with National Cholesterol Education Program Adult Treatment Panel III, but not IDF and Joint Interim Statement (JIS) criteria for MetS^16^.

Having a higher dietary acid–base load may increase the risk of MetS through multiple biological mechanisms. The diet with a high DAL score is characterized by a low intake of vegetables and fruit and higher intakes of dairy products, grains, and meat^21^. Fruits and vegetables have excellent antioxidant content that helps eliminate free radicals and prevent oxidative stress^31^; in contrast, meat consumption is considered to be positively associated with promoting inflammatory markers^32^. So, the high dietary acid–base load may lead to a prooxidant/antioxidant imbalance and chronic inflammation. Oxidative stress has a vital role in the pathogenesis of MetS. Oxidative stress can cause disturbances in the active sites of enzymes and eventually lead to disruptions in cell signalling and the state of metabolism^33^. Another mechanism linking high DAL to the risk of metabolic syndrome is increased cortisol secretion in the presence of metabolic acidosis. Hypercortisolism is considered one of the mechanisms involved in developing MetS by inhibiting insulin action, increasing lipase activity and gluconeogenesis^34–36^. By the way, utilizing alkaline reserves to neutralize acidity leads to mineral imbalances^12,37^, which may contribute to the disruption in insulin activity, lipid metabolism and blood pressure control^13,38^. Further studies are needed to elucidate the underlying biological mechanism linking dietary acid load to MetS.

This study was the first to examine the association of three different indicators of dietary acid–base load (DAL, NEAP, and PRAL) and the odds of metabolic syndrome in the general population of Iran, considering gender differences. However, some limitations must be expressed. Due to the study's cross-sectional design, we cannot infer a cause-effect relationships between nutrient patterns and the odds of metabolic syndrome. Besides, although a single 24-h dietary recall can be used to assess dietary intakes of the population, there are better instruments to investigate the association of diet as an independent variable with health outcomes. Also, due to the need for national food composition tables, misclassification of participants in terms of exposure might have occurred. Besides, past medical history and medications were assessed based on self-reported questionnaires, which may lead to the misclassification of study participants regarding the outcome. It should also be considered that our data did not include adhering to a special diet or taking supplements. In conclusion, we have found a significant positive association between three different indicators of dietary acid–base load and odds of metabolic syndrome among Iranian adults.

## Data Availability

The datasets generated and/or analyzed during the current study are not publicly available due to university data ownership policies, but are available from the corresponding author on reasonable request.
